# *Streptococcus infantis, Streptococcus mitis*, and *Streptococcus oralis* Strains With Highly Similar *cps5* Loci and Antigenic Relatedness to Serotype 5 Pneumococci

**DOI:** 10.3389/fmicb.2018.03199

**Published:** 2019-01-08

**Authors:** Fabiana Pimenta, Robert E. Gertz, So Hee Park, Ellie Kim, Iaci Moura, Jennifer Milucky, Nadine Rouphael, Monica M. Farley, Lee H. Harrison, Nancy M. Bennett, Godfrey Bigogo, Daniel R. Feikin, Robert Breiman, Fernanda C. Lessa, Cynthia G. Whitney, Gowrisankar Rajam, Jarad Schiffer, Maria da Gloria Carvalho, Bernard Beall

**Affiliations:** ^1^Division of Bacterial Diseases, National Center for Immunization and Respiratory Diseases, Centers for Disease Control and Prevention, Atlanta, GA, United States; ^2^Department of Medicine, Emory University School of Medicine, Atlanta, GA, United States; ^3^Atlanta Veterans Affairs Medical Center, Atlanta GA, United States; ^4^Johns Hopkins Bloomberg School of Public Health, Baltimore, MD, United States; ^5^Department of Medicine, University of Rochester School of Medicine and Dentistry, Rochester, NY, United States; ^6^Kenya Medical Research Institute, Nairobi, Kenya; ^7^International Emerging Infections Program, Centers for Disease Control and Prevention, Nairobi, Kenya

**Keywords:** capsular serotype, capsular biosynthetic, opsonophagocytic killing (OPK), immunodiffusion analysis, serotype-specific gene

## Abstract

*Streptococcus pneumoniae* is a highly impactful bacterial pathogen on a global scale. The principal pneumococcal virulence factor and target of effective vaccines is its polysaccharide capsule, of which there are many structurally distinct forms. Here, we describe four distinct strains of three Mitis group commensal species (*Streptococcus infantis*, *Streptococcus mitis*, and *Streptococcus oralis*) recovered from upper respiratory tract specimens from adults in Kenya and the United States that were PCR-positive for the pneumococcal serotype 5 specific gene, *wzy5*. For each of the four strains, the 15 genes comprising the capsular polysaccharide biosynthetic gene cluster (*cps5*) shared the same order found in serotype 5 pneumococci, and each of the serotype 5-specific genes from the serotype 5 pneumococcal reference strain shared 76–99% sequence identity with the non-pneumococcal counterparts. Double-diffusion experiments demonstrated specific reactivity of the non-pneumococcal strains with pneumococcal serotype 5 typing sera. Antiserum raised against *S. mitis* strain KE67013 specifically reacted with serotype 5 pneumococci for a positive Quellung reaction and stimulated serotype 5 specific opsonophagocytic killing of pneumococci. Four additional commensal strains, identified using PCR serotyping assays on pharyngeal specimens, revealed loci highly homologous to those of pneumococci of serotypes 12F, 15A, 18C, and 33F. These data, in particular the species and strain diversity shown for serotype 5, highlight the existence of a broad non-pneumococcal species reservoir in the upper respiratory tract for the expression of capsular polysaccharides that are structurally related or identical to those corresponding to epidemiologically significant serotypes. Very little is known about the genetic and antigenic capsular diversity among the vast array of commensal streptococcal strains that represent multiple diverse species. The discovery of serotype 5 strains within three different commensal species suggests that extensive capsular serologic overlap exists between pneumococci and other members of the diverse Mitis group. These findings may have implications for our current understanding of naturally acquired immunity to *S. pneumoniae* and pneumococcal serotype distributions in different global regions. Further characterization of commensal strains carrying homologs of serotype-specific genes previously thought to be specific for pneumococci of known serotypes may shed light on the evolution of these important loci.

## Introduction

Serotype 5 is one of more than 91 pneumococcal capsular serotypes (Geno et al., 2015) and is included in current pneumococcal conjugate vaccine formulations (13- and 10-valent) and the 23-valent polysaccharide vaccine. Like serotype 1, serotype 5 is one of a limited number of serotypes that are highly associated with invasive pneumococcal disease (IPD) and localized disease clusters compared to most other pneumococcal serotypes (Hausdorff, 2007; Romney et al., 2008; Balicer et al., 2010; Tomczyk et al., 2016). Serotypes 1 and 5 were found to have the highest “invasive index” among pneumococci, based upon their relatively high propensity to be recovered from IPD rather than from carriage samples (Brueggemann et al., 2004).

In a 2009 carriage study in Kenya (Carvalho et al., 2013; Conklin et al., 2016) in an area with high pneumococcal carriage among adults (median age 32 years; 43.2 and 26.8% frequency for pneumococcal isolation from combined oropharyngeal and nasopharyngeal specimens from HIV-positive and HIV-negative adults, respectively), we found that a very high percentage of specimens negative for a pneumococcal-specific *lytA* PCR assay yielded pneumococcal serotype-specific PCR amplicons (Carvalho et al., 2013). The sequences of these amplicons were highly homologous to the serotype-specific pneumococcal counterparts leading us to speculate that it was likely that such strains expressed surface carbohydrates highly related to those expressed from different serotypes of pneumococci. For several of *wzy*-positive, *lytA*-negative specimens we were able to recover non-pneumococcal Mitis group streptococci, including strains that were positive for PCR assays (Pai et al., 2006; Pimenta et al., 2013) targeting *wzy5*, *wzy12F*, *wzy15A*, and *wzy33F.* We found that a high percentage of upper respiratory specimens from adults were positive for the serotype 5 specific *wzy5* gene when employing a conventional PCR assay (Pai et al., 2006) (32.3%; 51 of 158 specimens), even though we were unable to recover serotype 5 pneumococcal isolates from specimens taken from adults (Carvalho et al., 2013). Subsequently, employing standard culture methodology we readily isolated a *Streptococcus mitis* strain that was PCR-positive for the serotype 5-specific *wzy5* gene (strain KE67013 shown in Table 1) from these specimens. In another carriage study performed in the United States during 2015–2016 in a population of older adults (age ≥ 65 years) with much lower pneumococcal carriage (1.2% by culture), we also found a relatively high number of *wzy5*-positive upper respiratory specimens from *lytA*-negative (indicative of pneumococcal-negative) specimens (11/395, 2.8%) and a single *wyz5*-positive from a less common *lytA*-positive specimen (1/53, 1.9%) (Lessa et al., 2018). Importantly, our recent study (Lessa et al., 2018) described the isolation of five independent *Streptococcus mitis* strains representing three unrelated clones that expressed a capsular serotype highly related to serotype 1 pneumococci as deduced from immunodiffusion experiments and opsonophagocytic killing (OPK) assays. What is most noteworthy from this study was that the frequency of carriage of capsular serotype 1 non-pneumococcal isolates within this elderly population was actually much higher than the carriage of serotype 1 pneumococci.

**Table 1:** Non-pneumococcal strains carrying *cps* locus homologs of pneumococcal strains of known serotypes described in this manuscript.

					Capsular serology result	OPK activity of
				Source of	employing pneumococcal	rabbit antiserum
Strain	Year and country of origin	Species	*cps* homolog	isolate^c^	typing sera	against strain
KE67013	2009 Kenya^a^	*S. mitis*	5	Adult OP	Serotype 5	Specific killing of type 5
						pneumococci
US0049	2013 United States^b^	*S. oralis*	5	Adult OP	Serotype 5	Not tested
US0024	2013 United States^b^	*S. infantis*	5	Adult OP	Serotype 5	Not tested
US969j1	2013 United States^b^	*S. infantis*	5	Adult OP	Serotype 5	Not tested
KE67213	2009 Kenya^a^	*S. oralis*	12F	Adult OP	Serogroup 12	Not tested
KE66813	2009 Kenya^a^	*S. oralis*	15A	Adult OP	No reactivity	Not tested
KE66713	2009 Kenya^a^	*S. mitis*	18A	Child NP	Serotype 18A (weak)	Not tested
KE66913	2009 Kenya^a^	*S. oralis*	33F	Adult OP	Serogroup 33 (weak)	Not tested

^a^*Isolation previously described (Carvalho et al., 2013); details of carriage study previously described (Conklin et al., 2016). ^*b*^Isolation from carriage specimens carried out in this work from carriage specimens described in year 2015–2016 carriage study (Lessa et al., 2018). ^*c*^OP = oropharyngeal swab specimen; NP = nasopharyngeal swab specimen. Only one specimen was from a child <5 years of age*.

While, we were also unable to isolate serotype 5 pneumococci in the US study (Lessa et al., 2018), we have subsequently isolated two distinct strains of *wzy5*-positive *S. infantis* and one *S. oralis* strain from *lytA*-negative specimens. Our genomic and serologic analysis of these four strains from three distinct Mitis group species that carry *cps5* loci recovered from upper respiratory specimens described in two previously described carriage studies (Carvalho et al., 2013 and Conklin et al., 2016 for the single *S. mitis* strain; Lessa et al., 2018 for a single *S. oralis* strain and two *S. infantis* strains) extends recent descriptions of several *cps* loci from non-pneumococcal Mitis group streptococci that shared high genetic and antigenic relatedness to known pneumococcal serotypes (Skov Sørensen et al., 2016). Here, we extend our recent work that demonstrated cross-species OPK activity of serotype 1 *S. mitis* antiserum against serotype 1 pneumococci (Lessa et al., 2018), through demonstration of serotype-specific opsonic activity of *wzy5*-positive *S. mitis* antiserum against serotype 5 pneumococci. We also describe genomic and serologic features of four additional non-pneumococcal strains previously recovered in Kenya (Carvalho et al., 2013) that contain homologs of serogroup 12, 15, 18, and 33 pneumococcal *cps* loci.

The growing evidence of conserved capsular serotypes between the commensal Mitis group and pneumococci, including recently published findings (Carvalho et al., 2013, Skov Sørensen et al., 2016; Lessa et al., 2018) and the work presented here, may eventually shed light on the past and present distributions of epidemiologically important pneumococcal serotypes.

## General Genomic Features of Non-Pneumococcal Strains Carrying *cps* Loci Closely Similar to Serotype 5 Pneumococci

Genome sequences of the 4 *wzy5*-positive isolates revealed that all lacked the pneumococcal-specific *piaA* iron transporter determinant (Whalan et al., 2006). Sequence from strain F0392, for which only the genome sequence has been available but corresponding strain serology not characterized (Skov Sørensen et al., 2016), is also included for comparison. Although negative for the pneumococcal *lytA* PCR assay Carvalho et al. (2007) Strain KE67013 contained recognizable homologs of the major pneumococcal autolysin (*lytA*) and of the pneumolysin gene (*ply*), with 79 and 60% sequence identity, respectively. Strain F0392 contained a *lytA* homolog (72% identity), while strains US0049, US969j1, and US0024h lacked recognizable homologs of all three genes.

**FIGURE 1:**
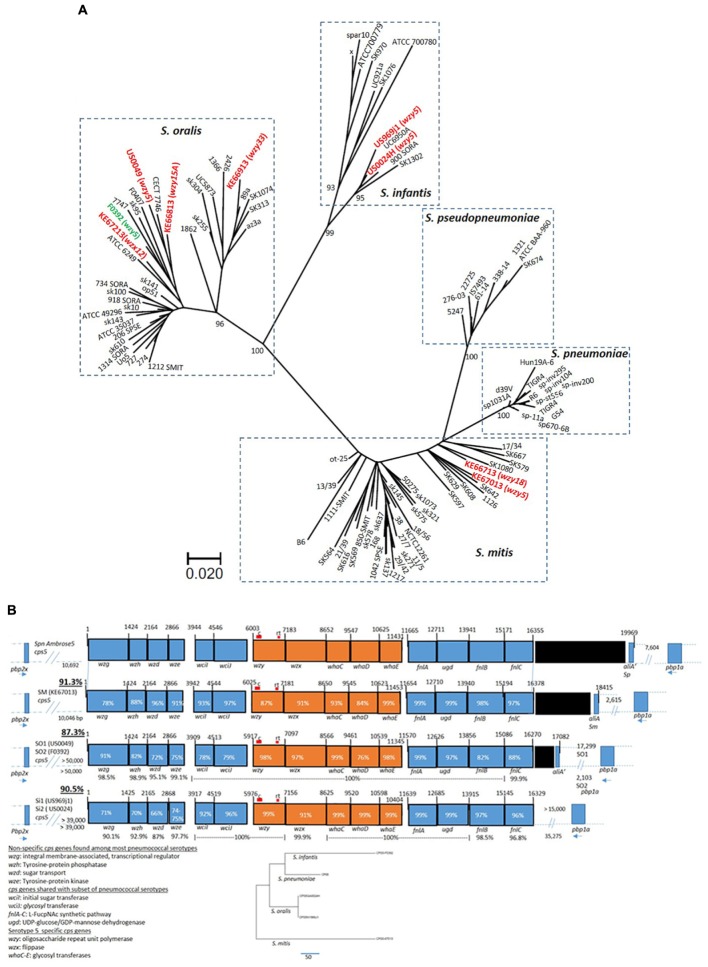
**(A)** Maximum parsimony tree based upon kSNP3.0 analysis (Gardner et al., 2015) of eight study genomes (indicated in red including *wzy* or *wzx* genes initially detected in PCR-serotyping assay) from this study, combined with 66 genomes from GenBank within the species *S. mitis*, *S. oralis*, and *S. infantis* described previously (Jensen et al., 2016). The k-mer size employed was 19. The scale is based upon 322 core SNPs. The indicated node support values were calculated by FastTreeMP as described (Gardner et al., 2015). The genome from strain F0392 obtained from NCBI (indicated in green) is also included. **(B)** Alignments of *cps5* operons from five strains of non-pneumococcal species *S. mitis* (SM) 67013, *S. oralis* (SO, strains US0049 and F0392), and *S. infantis* (SI; strains US969j1 and US0024). The percent sequence identity of the entire biosynthetic cluster (*wzg* through *fnlC*) with the pneumococcal serotype 5 reference sequence (GenBank accession CR931637) is underlined at left. The percent identity of the allele over its entire overlap with the pneumococcal reference is indicated within each rectangle representing the indicated gene. For the *S. oralis* and *S. infantis* alignments that each depict a pair of distinct strains, each gene has the indicated conserved translational start and/or end. *S. mitis* 67013 is the only strain showing close linkage of the *cps5* locus with upstream *pbp2x* and downstream *pbp1a*, while the two *S. oralis cps5* loci lie 2–17 kb upstream of *pbp1a*. The position of conventional (c) and real time (rt) serotype 5 detection assays are indicated (c assay positive for all four strains tested, rt assay positive for all but *S. mitis* US67013). The percent identities are indicated between the two pairs of strains (US0049/F0392 and US969j1/US0024) below the indicated genes. The five *cps5* genes that appear entirely serotype 5-specific (<54% identical to all other known pneumococcal *cps* genes) are indicated in orange. Phylogenetic analysis shows the relative relatedness of the five serotype 5 specific genes between the four species. Gene functions listed at bottom left are taken from accession CR931637. Black rectangles indicate transposase gene remnants. The orientations of *pbp2x* and *pbp1a* relative to *cps5* are indicated at the right and left end, respectively, of each *cps5* operon. The coordinates above the genes indicate base pairs (bp), also indicated as distances between the *cps* loci and flanking *pbp* genes.

Phylogenetic analysis employing kSNP3.0 (Gardner et al., 2015) revealed that the five *wzy5*-positive strains [including strain F0392 (Skov Sørensen et al., 2016)] were genetically highly diverse, clustering with representatives of *S. infantis*, *S. mitis*, and *S. oralis* clusters (Figure 1A). Phylogenetic analysis employing concatenated housekeeping gene fragments as previously described (Bishop et al., 2009) was in close agreement with the depicted whole genomic kSNP.0 analysis (Supplementary Figure S1). For the non-study strains depicted in Figure 1A, kSNP3.0 analysis (Figure 1A) was also generally in agreement with their previous genomic-based species assignments (Jensen et al., 2016).

All five non-pneumococcal *wzy5*-positive strains contained *cps* loci highly similar to the corresponding *cps5* locus from the serotype 5 pneumococcal reference strain, with 87.3– 90.5% sequence identity over the entire operon (Figure 1B). Highly homologous (78–99.2% sequence identity) counterparts of each of the previously described 15 *cps5* genes from the pneumococcal serotype 5 reference strain were apparent in the same relative order. The first four genes of the *cps5* locus (*wzg*, *wzh*, *wzd*, and *wze*) have widely conserved functions in pneumococcal capsular polysaccharide synthesis, corresponding with high similarity (>98% sequence identity, regardless of serotype) to a large number of counterparts from pneumococcal *cps* operons (Mavroidi et al., 2007). An additional 6 *cps5* genes with more specialized functions are conserved among a subset of pneumococcal serotypes (Figure 1B). The centrally situated five *cps5* genes, including the *wzy* and *wzx* genes that encode highly substrate-specific flippase and polymerase functions, share little or no sequence similarity with pneumococcal strains of other known capsular serotypes; however, these pneumococcal genes shared 76–99% sequence identity among the five non-pneumococcal strains shown. The highly conserved five-gene segment was exactly 5,429 bases in length in all six strains (including *S. pneumoniae* strain Ambrose) and shared the same spacings of translational start and stop codons.

The presence of the *fnlA-C* genes in serotype 5 (Figure 1B) is consistent with N-acetyl-α-L-fucosamine found within serotype 4, 5, 12A, and 12F capsule polysaccharides (Kamerling, 2000; Mavroidi et al., 2007). Further, N-acetyl-L-pneumosamine and 4-keto-N-acetyle-D-quinovosamine, intermediates within the N-acetyl-α-L-fucosamine pathway, are both uniquely present in the pneumococcal serotype 5 capsule. As previously speculated (Mavroidi et al., 2007), the marked sequence divergence of the *cps5 fnlA* gene compared to *fnlA* alleles of types 4, 12A, and 12F might be related to the unique final products produced in the *cps5* N-acetyl-α-L fucosamine pathway. The near-identical *fnlA* sequences of the five *wzy5*-positive commensal strains of the three different species and serotype 5 pneumococci are consistent with structural similarity or identity between the capsular polysaccharides (Figure 1B).

One or both of the *pbp2x* and *pbp1a* genes are sometimes co-transferred along with *cps* loci during pneumococcal gene replacement events (Brueggemann et al., 2007; Wyres et al., 2013). The location of *cps5* in the genome relative to *pbp1a* and *pbp2x* varied between the commensal strains (Figure 1B), although the *S. mitis cps5* locus showed the same orientation and relative genomic location between the upstream *pbp2x* and downstream *pbp1a* genes as the serotype 5 pneumococcal reference strain. The two *S. oralis cps5* loci were not closely linked to *pbp2x*, but linkage to the downstream convergent *pbp1a* gene was observed. Genomic proximity of *cps5* from the two *S. infantis* strains to *pbp2x* was also lacking, however, *cps5* from one of the two strains (US0024h) was situated 35 kb upstream of the convergent *pbp1a*. Due to the smaller length of the *cps5*-containing contig, we were unable to verify similar linkage of *cps5* from *S. infantis* US0969j1 to *pbp1a*.

As with pneumococcal *cps* loci, the *cps5* locus from *S. mitis* KE67013 and the two *S. oralis* strains were situated between *dexB* and *amiA*. The *cps5* loci from the two *S. infantis* strains isolated within the United States differed in their genomic location compared to the other three species in that it was situated immediately upstream of the cell division gene *ftsA* (not shown).

## Shared Serospecificity of Non-Pneumococcal *cps5*-Carrying Strains With Serotype 5 *S. pneumoniae*

**FIGURE 2:**
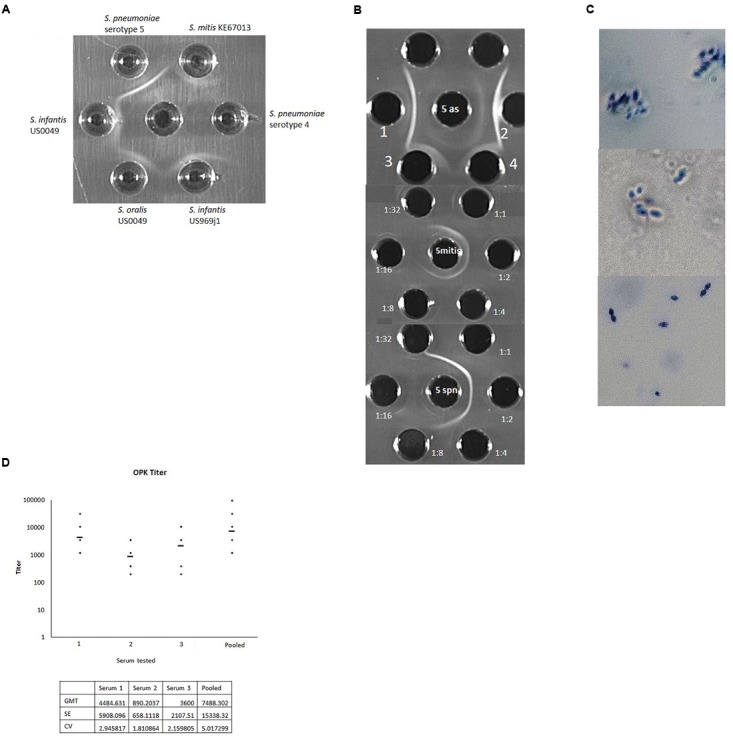
**(A)** Reactivity of typing antisera raised against serotype 5 pneumococcal strain Ambrose (middle well) against *wzy5*-positive strains of *S. infantis*, *S. oralis*, *S. mitis*, and serotype 5 *S. pneumoniae* Ambrose. A serotype 4 pneumococcal strain is included as a negative control. In other experiments pneumococcal serotype 1 and *S. mitis cps1*-positive strain L006 (Lessa et al., 2018) were included as negative controls and showed no reactivity. **(B)** Top: Reactivity of pneumococcal serotype 5 typing antisera against *S. mitis* KE67013 (top grid, center well contains type 5 antisera; wells 1 and 2 contain type 5 pneumococcal extract. Wells 3 and 4 contain strain KE67013 extract. Middle and lower grids show reactivity of indicated dilutions of pneumococcal typing antisera (peripheral wells) against *S. mitis* KE67013 extract (middle grid, center well) and serotype 5 *S. pneumoniae* strain Ambrose extract (lower grid, center well). **(C)** Positive Quellung reactions of serotype 5 pneumococcal strain Ambrose when reacted with antisera raised against *wzy5*-positive *S. mitis* strain KE67013 (top panel) and with standard serotype 5 typing antiserum (middle panel). The same pneumococcal type 5 strain showed no reactivity when exposed to antisera prepared against an *S. mitis* strain L006 containing a *cps1*-like operon (Lessa et al., 2018) unrelated to *cps5* (bottom panel). **(D)** Opsonophagocytic killing (OPK) activity of rabbit antisera raised against serotype 5 *S. mitis* KE67013 in three separate rabbits (1–3) and of pooled, clarified antisera from the three rabbits (pooled). Geometric Mean Titer (GMT) values of titers across 5–6 assay runs are shown with >50% killing compared with the growth in the complement control wells. Initial dilution was 1:400 with subsequent two-fold dilutions down to 1:51200. Test of the rabbit antiserum generated against serotype 5 *S. pneumoniae* gave optimal titer at 1:960 (not shown). KE67013 was included as the positive control in the OPK assay; KE67013 antiserum had very high (titer > 3000) opsonic activity against strain KE67013 (not shown). Pre-inoculation bleeding samples for all rabbits induced no killing (data not shown). Antisera prepared against control strain of *S. mitis* L006 carrying a full-length *cps1*-like operon (strain and antiserum described in Lessa et al., 2018) unrelated to *cps5* showed no OPK activity against serotype 5 pneumococci. Coefficient of variation (CV) = standard deviation/GMT;SE (*σ*) = GMT ^∗^ (exp (standard deviation) – 1)/sqrt (N) $\sigma =\sqrt{\frac{1}{N}\sum_{i=1}^{N}{\left(X_{i}-\mu \right)}^{2}.}$ Where, *x_i_* is an individual natural-log transformed value *μ* is the mean/expected value and *N* is the total number of values. $\text{CV}=\sqrt{e^{\sigma^{2}}-1}.$

For unknown reasons we could not see a positive Quellung reaction for three of the four *cps5*-positive non-pneumococcal strains with pneumococcal typing serum specific for serotype 5, however, we could detect a subtle positive result for *cps5*-positive *S. mitis* strain KE67013 (data not shown). Immunodiffusion experiments demonstrated specific reactivity of each strain with anti-pneumococcal type 5 typing serum (Figure 2A,B).

In addition, antiserum produced against strain KE67013, the strain which yielded the weakest immunodiffusion results with anti-pneumococcal type 5 serum (Figure 2A), exhibited strong specific reactivity with pneumococcal serotype 5 strains in immunodiffusion (not shown) and in the Quellung reaction (Figure 2C).

This same antiserum raised against strain KE67013 was highly and specifically active in opsonophagocytosis killing (OPK) assays directed against serotype 5 pneumococci (Figure 2D), and showed no OPK activity against pneumococci of serotypes 1 and 4 (data not shown). The OPK activity of 3 of the 4 antiserum samples (1st, 3rd, and pooled depicted in Figure 2D) against serotype 5 *S. pneumoniae* was actually higher than our typing antisera prepared against type 5 *S. pneumoniae* (Figure 2D). Additionally, antisera prepared against a control strain of *S. mitis* carrying a full-length *cps1*-like operon (strain and antiserum described in Lessa et al., 2018) unrelated to *cps5* showed no OPK activity against serotype 5 pneumococci.

## Genetic and Serologic Features of Other Non-Pneumococcal Strains With Additional Pneumococcal-Like *cps* Loci

Included in Figure 1A and Table 1 are non-pneumococcal strains recovered in samples from the same Kenya study (Carvalho et al., 2013) that were PCR-positive for pneumococcal serotypes other than serotype 5 and were assigned non-pneumococcal species based upon housekeeping gene sequence phylogeny (Bishop et al., 2009, Supplementary Figure S1) and WGS-based phylogeny (Figure 1A). These serogroups included 18 (assigned as *S. mitis* KE66713), 15A/15F (*S. oralis* KE66813), 33 (*S. oralis* KE66913, and 12 (*S. oralis* KE67213). When comparing the genomic locations of *cps* operons from *S. mitis* (*cps18*) and *S. oralis* (*cps15A*, *cps33F*, and *cps12F*) with *cps* loci highly similar to those found in pneumococci of the same specific serotypes (Supplementary Figure S2), only *cps18* from *S. mitis* KE66713 showed close linkage to both *pbp2x* and *pbp1a* (data not shown). *S. mitis* KE66713 (*cps18*) was the only commensal isolate described here that was from a child and was recovered from a nasopharyngeal specimen (Carvalho et al., 2013).

Immunodiffusion experiments showed specific reactivity of *S. mitis* strain KE66713 indicative of being weakly positive for serotype 18A (reactivity with serotyping factor 18d). *S. oralis* KE66913 (*cps33*) showed pool E reactivity (pool E consists of all factors for resolution of serogroups 12 and 33, along with serotypes 13, 44, and 46) which could only be narrowed to very weak serogroup 33 reactivity. Similarly, KE67213 (*cps12*) demonstrated strong reactivity with pool E, but the reactivity could only be narrowed to serogroup 12, due to no reactivity with individual serogroup 12 factors. We did not observe detectable *S. oralis* strain KE66813 (*cps15A*) reactivity with pneumococcal serogroup 15 antiserum.

## Resistance Features Found Within Non-Pneumococcal Strains Carrying *cps* Locus Homologs of Known Pneumococcal *cps* Loci

It was interesting to find that six of the seven non-pneumococcal strains included within this study and all five of the cps1-positive *S. mitis* strains from the previous study were non-susceptible to one or more antibiotics, with these resistance features corresponding to determinants detected through our pneumococcal WGS bioinformatics pipeline (Table 2). Nine of the 13 isolates were non-susceptible to one or both of the beta lactam antibiotics penicillin or ceftriaxone. It was interesting that the pneumococcal PBP typing scheme identified one of five new PBP types (Metcalf et al., 2016a) for six of the seven *S. mitis* strains. For all other strains, 1–2 of the 3 PBP subtypes were not generated since they carried divergent PBP gene alleles that were not identifiable within the current pipeline parameters. All seven of the PBP typed *S. mitis* strains that carried one of five new PBP types. Conventionally determined MICs for penicillin and ceftriaxone were in close agreement with a machine learning based algorithm that predicts beta lactam MICs for new PBP types (Li et al., 2017). Two highly related *cps1*-positive *S. mitis* isolates (L115 and L116) shared the same PBP types that included a penicillin binding protein subtype (2*x* – 8) commonly found within multiple penicillin-non-susceptible pneumococcal clones (Beall et al., 2018). Six isolates carried macrolide resistance determinants (*mef/msrD* and/or *ermB*) that correlated to observed resistance for erythromycin and/or clindamycin. Eight isolates carried mutations within *folA* and/or *folP* genes that correlated to intermediate resistance (1–2 μg/ml) or full resistance (>4 μg/ml) to cotrimoxazole. Seven isolates contained *tetM* derivatives also predicting non-susceptibility to tetracycline. All three isolates that were susceptible to beta lactam antibiotics would not be considered to be basally susceptible as defined for basally susceptible pneumococci which uniformly have MICs < 0.03 μg/ml for both penicillin and ceftriaxone.

**Table 2:** Pneumococcal resistance features^A^ found within non-pneumococcal strains carrying homologs of pneumococcal *cps* loci.

Non-pneumococcal
Isolate (*cps* type)	species	MICs^B^ (μg/ml)		other resistance determinants^A^	MICs^B^			
		Pen	Cft		ery	cli	cot	tet
KE67013 (5)	*S. mitis*	=0.5	= 0.25	FolA(I100L)/FolPins181, *tetM*	≤0.03	≤ 0.03	=4	> 8
US0049 (5)	*S. oralis*	≤0.03	= 0.12	negative	≤0.03	≤ 0.03	≤0.12	≤ 0.25
US0024 (5)	*S. infantis*	=0.5	=0.5	*mef/msrD*	=2	≤0.03	≤ 0.12	= 0.5
US969j1 (5)	*S. infantis*	= 0.06	= 0.06	negative	≤0.03	≤ 0.03	= 0.25	= 0.5
KE67213 (12)	*S. oralis*	=0.25	= 0.25	FolA(I100L), *tetM*	≤0.03	≤ 0.03	=2	= 4
KE66813 (15A/F)	*S. oralis*	=0.5	=0.5	FolA(I100L)	≤0.03	= 0.06	=4	= 0.5
KE66913 (33)	*S. oralis*	= 0.06	= 0.12	FolA(I100L)	≤0.03	≤ 0.03	=4	= 0.5
KE66713 (18)	*S. mitis*	=0.12	= 0.12	FolA(I100L)/FolPins181	≤0.03	≤ 0.03	=4	= 0.5
L006 (1)^C,D^	*S. mitis*	=1	=1	*ermB*, FolA (I100L), *tetM*	> 32	> 2	=4	= 4
L164 (1)^C,D^	*S. mitis*	=2	=1	*ermB*, FolA (I100L), *tetM*	> 32	> 2	=2	= 8
L115 (1)^C,D^	*S. mitis*	=2	=1	*mef/msrD*, FolA(I100L)/FolPins178, *tetM*	= 4	≤0.03	=4	> 8
L116 (1)^C,D^	*S. mitis*	=2	=1	*mef/msrD*, FolA(I100L)/FolPins178, *tetM*	= 4	≤0.03	=4	> 8
L121 (1)^C^	*S. mitis*	=0.12	= 0.06	*ermB, mef/msrD, tetM*	> 32	> 2	≤0.12	> 8

^A^*WGS pipeline features and abbreviations of resistance determinants as described (Metcalf et al., 2016a,b). ^*B*^Minimum inhibitory concentrations determined conventionally and MICs indicated in red are indicative for non-susceptibility within pneumococci according to previously described parameters (Metcalf et al., 2016a). pen, penicillin; ery, erythromycin; cli, clindamycin; cot, cotrimoxazole; tet, tetracycline. ^*C*^These five strains have been previously described (Lessa et al., 2018) and include two separate pairs of highly related strains (each indicated in blue or green font). These strains were recovered from the same US carriage study as strains US0049, US0024, and US969j1 that are included in this study and table. ^*D*^The two highly related isolates L115 and L116 shared identical PBP types with both contained penicillin binding protein subtype PBP2X-8 commonly found within penicillin-non-susceptible pneumococci (Metcalf et al., 2016b; Beall et al., 2018). Highly related strains L006 and L164 shared 2 of the 3 PBP subtypes that define the PBP type. Of the 7 *S. mitis* included, only L121 did not yield an identifiable PBP type through the pneumococcal WGS resistance pipeline*.

## Discussion

What is known about directionality of interspecies horizontal gene transfer between pneumococci and other Mitis group streptococcal species has shown that most observed transfer has been from commensal species donors to pneumococcal recipients (Dowson et al., 1993; Sibold et al., 1994; Kilian et al., 2014). Much of this directionality has been shown through sequences of resistance-conferring PBP gene alleles, which invariably reveal that resistant pneumococcal clinical and carriage isolates contain PBP gene sequences that had their origins within non-pneumococcal Mitis group streptococci that are generally either *S. mitis* or *S. oralis* (Dowson et al., 1993; Sibold et al., 1994). This could be causally related to the higher abundance and longer carriage duration of commensal Mitis group species within the human host compared to pneumococcal strains, providing a vast commensal Mitis group donor gene pool. While we believe that it would not be surprising if non-pneumococcal species have been the source of individual serotype-specific *cps* genes (Kilian et al., 2014), we believe that it is presently difficult to formally prove their origins based upon the paucity of individual sequenced serotype-specific alleles from commensal species. There is a very high level of genetic diversity observed between the non-pneumococcal species and/or strains carrying *cps5*-like loci [the 4 strains (3 species) described in this work and the type 5 *S. oralis* genome from Skov Sørensen et al., 2016] and *cps1* loci [3 unrelated clones (5 isolates) of *S. mitis* described by Lessa et al., 2018]. In view of present available data, serotype 1 and serotype 5 specific genes may well have been transferred from pneumococci to Mitis group species, or even have been derived from an undiscovered or extinct species (Lessa et al., 2018). In pneumococci, two of the three key PBP genes closely flank the *cps* locus. Therefore, it is not surprising that *pbp2x* and *pbp1a* alleles have often been found to be co-transferred during recombinational *cps* locus replacement events (Brueggemann et al., 2007; Wyres et al., 2013; Metcalf et al., 2016b; Beall et al., 2018). In general, however, the intraspecies conservation of pneumococci *cps* loci combined with the relative conservation of non-PBP genes flanking pneumococcal *cps* loci transferred during serotype-switch events (unpublished and GenBank data) seems to indicate that commensal species rarely serve as genetic donors of *cps* loci found within disease-causing pneumococcal strains.

From recent observations (Carvalho et al., 2013; Skov Sørensen et al., 2016; Lessa et al., 2018) and what we have observed here from a limited sampling of recently isolated naturally occurring commensal streptococci, the extent of highly conserved capsular biosynthetic loci shared between the global pathogen *S. pneumoniae* and other related members of the streptococcal Mitis group is far-reaching. Conjugate vaccines targeting pneumococcal serotypes decreases colonization of vaccine serotype pneumococci in the upper respiratory tract, and studies indicate that pneumococci have decreased expression of capsule during carriage relative to infection (Hammerschmidt et al., 2005). Very little is known regarding the extent of expression of capsular polysaccharides by related non-pneumococcal species. Serotype 5 invasive disease has been rare in the United States even before the introduction of conjugate vaccines (Pilishvili et al., 2010). The relative rarity of pneumococcal type 5 strains in previous carriage studies undertaken in Kenya (Conklin et al., 2016) and in the United States (Sharma et al., 2013; Desai et al., 2015), could be reflective of cross-species immunity conferred through the expression of capsule from commonly carried commensal serotype 5 strains of at least three different species in the upper respiratory tract. This possibility is speculative and requires additional studies, including those that assess specific immunologic responses from carriers of serotype 5 commensal streptococci. There are other potential reasons for the scarcity of serotype 1 and 5 strains causing disease within the past several decades. Pneumococci of both serotypes 1 and 5 are quite clonally restricted and are generally susceptible to antimicrobial agents, with extreme susceptibility to beta lactam antibiotics. It is possible that the broad usage of penicillin and other antibiotics in the later part of the 20th century played significant roles in the marked decrease of both serotypes, both of which were responsible for a heavy disease burden in the United States earlier in the century (Heffron and Varley, 1932). The five serotype 1 and three of the serotype 5 non-pneumococcal strains examined here had more resistance determinants and higher MICs to different antibiotics than typical pneumococcal isolates of these same serotypes recovered from different geographic regions (Hausdorff, 2007; Romney et al., 2008; Balicer et al., 2010; Metcalf et al., 2016a; Tomczyk et al., 2016). These resistance features, including higher MICs to beta lactams, are likely to provide an advantage for their persistence within the upper respiratory tract carriage reservoir relative to pneumococcal strains of these same serotypes.

The findings that non-pneumococcal isolates of serotypes 1 and 5 are carried within the elderly US population at higher frequencies than pneumococci of these same serotypes presents a new landscape for the study of natural occurring pneumococcal immunity and serotype distributions (Carvalho et al., 2013; Lessa et al., 2018). Similarly, it appears likely that serotype 5 *S. mitis* occurred within the Kenya carriage survey at a higher frequency than serotype 5 pneumococci (Carvalho et al., 2013; Conklin et al., 2016). It will also be interesting to learn whether commensal strains of serotypes 1, 5 and other vaccine serotypes colonize different age groups with similar efficiencies within vaccinated and unvaccinated populations; in the previous sampling of older US adults, those who had received PCV13 were less likely than unvaccinated adults to be PCR-positive for genes specific for PCV13-serotypes within specimens PCR-negative for pneumococci (Lessa et al., 2018).

### Bacterial Strains

Centers for Disease Control and Prevention (CDC) and local institutional review boards approved the studies (CDC protocol #6725). The Kenya study was approved by ethics committees at the Kenya Medical Research Institute and CDC as described (Carvalho et al., 2013; Conklin et al., 2016). Methods for isolation of the non-pneumococcal strains that were PCR-positive for serotype 5 and other known pneumococcal serotypes from upper respiratory specimens are as described (Carvalho et al., 2013). Conventional and real time PCR assays were previously described (9, 27) with updates posted at https://www.cdc.gov/streplab/pneumococcus/resources.html. The *cps5*-positive strains US0024, US969j1, and US0049 were recovered during this study from a stored US carriage study specimen collection (Lessa et al., 2018). The genome sequence of strain F0392 was obtained from GenBank project accession AFUO01.

### Genomic Sequencing

Genomic DNA samples from all isolates were prepared and sequenced as multiplexed libraries on the Illumina MiSeq platform to produce paired end reads (Metcalf et al., 2016b).

### Preparation of Streptococcal Antisera

This protocol (protocol number 2776GERRABC-A1) was approved by the Institutional Animal Care and Use Committee (IACUC). Antiserum against formalin-fixed *S. mitis* strain KE67013 was prepared exactly as previously described for CDC capsular typing antisera prepared against pneumococcal strains (Lund and Henrichsen, 1978). Three rabbits were inoculated over a period of six weeks to yield the three antiserum sources used. A pooled, chloroform clarified sample that combined all three sources was also used.

### Serology

Latex agglutination and the Quellung reaction employing rabbit polyclonal typing antiserum were used to assess serotype expression in commensal streptococci. Double immunodiffusion assays employing pneumococcal typing sera and antisera prepared against commensal streptococci were carried out as previously described (Skov Sørensen et al., 2016).

### Species Assignments

Strains were assigned species by virtue of clustering with previously speciated strains (Jensen et al., 2016) employing whole genomic kSNP3.0 analysis to generate core genomic single-nucleotide polymorphism and a maximum parsimony phylogenetic tree with the indicated node support as described (Gardner et al., 2015). Node support was assessed by using 500 bootstrap replicates. Phylogenetic clustering of concatenated housekeeping gene sequences (multilocus sequence analysis; MLSA) was achieved with the Mega7 program as previously described (Bishop et al., 2009).

### Opsonophagocytosis (OPK) Assays

The standard OPK assay was performed employing HL-60 cells and complement source (baby rabbit serum; Pel-Freez, Brown Deer, Wis.) as outlined previously (Romero-Steiner et al., 1997). Initial dilution was determined at 1:400 based on optimization testing with serotype 5 *S. pneumoniae*-induced antisera against type 5 *S. mitis* KE67013. Complement control wells included all the test reagents except antibodies to pneumococci. Opsonophagocytic titers taken for Geometric Mean Titer (GMT) reflect the serum dilution with >50% killing compared with the mean growth in the complement control wells.

## Genomic Fastq Accession Numbers

Accession numbers for KE66713–KE67213, US0024, US0049, and US969 fastQ files are SAMN09874918 through SAMN9874925 within BioProject PRJNA480039.

## Note Added in Proof

Genevieve Gariss and colleagues at the Karolinska Institutet have described an apparently serotype 5 strain of a fifth Mitis group species, *S. pseudopneumoniae*: see 10.1101/468462.

## Author Contributions

All authors listed have made a substantial, direct and intellectual contribution to the work, and approved it for publication. FP and MC isolated all Mitis group strains. RG performed immunodiffusion and Quellung assays. RG was responsible for preparation of all rabbit antisera. SP and EK performed OPK assays with direction of GR and JS who also interpreted the data. FP, NR, MF, GB, DF, RB, FC, CW, GW, JS, MC, and BB designed different aspects of the study. NR, NB, LH, MF, GB, DF, and RB oversaw activities at the study sites. BB was responsible for genomic analyses and oversight of lab activities. BB wrote the manuscript with input provided by all authors.

## Conflict of Interest Statement

The authors declare that the research was conducted in the absence of any commercial or financial relationships that could be construed as a potential conflict of interest.

## Supplementary Material

FIGURE S1 Assignment of study strains to species employing phylogenetic clustering of concatenated housekeeping genes as previously described (Bishop et al., 2009). Where not labeled the sequences were obtained from this study and shared the same species clustering. Methodology follows this paper exactly, except that only the four closest species clusters to *S. pneumoniae* were included. Twenty pneumococcal strains of 10 different serotypes were included. Established reference strains with genomes accessible in public databases include the indicated pneumococcal strains, ATCC BAA960 and IS7493 (*S. pseudopneumoniae*); NCTC 12261, B6, and SK578 (*S. mitis*); ATCC 700779, SK1302 (*S. infantis*); and CCECT7747, UO5, SK313, SK141, OD321121-09 (*S. oralis*). The optimal tree with the sum of branch length = 5152.37943912 is shown. The tree is drawn to scale, with branch lengths in the same units as those of the evolutionary distances used to infer the phylogenetic tree. The evolutionary distances are in the units of the number of base differences per sequence. The analysis involved 136 nucleotide sequences. Codon positions included were 1st+2nd+3rd+Non-coding. There were a total of 3063 positions in the final dataset for each strain.

FIGURE S2 General features of additional homologs of *cps* loci from non-pneumococcal species (SO, *S. oralis*; SM, *S. mitis*) compared to pneumococci of known capsular serotypes. The genes aligned by the slanted lines indicate the polysaccharide synthetic gene cluster. Within each gene of the cluster, the percent identity between the two homologs is shown. In each *cps* operon shown the gene cluster lies between *dexB* and *aliA*. White rectangles indicate open reading frames (orfs) that lack homology with the respective pneumococcal reference sequence. The red lines indicate region targeted by conventional and real time “PCR-serotyping” assays. The orange open reading frames share >60% sequence identity only with pneumococcal strains within small serogroups as follows: **A**, 12F/12A/12B/44/46; **B**, 15A/15F; **C**, 18C/18A/18B/18F; **D**, 33F/33A/37. Black rectangles do not represent open reading frames, but have spurious homology to transposase structural genes. For descriptions of conserved and serotype-specific gene functions, see the indicated GenBank accessions for the pneumococcal reference serotypes as described (Bentley et al., 2006; Aanensen et al., 2007; Mavroidi et al., 2007). **(A)** Comparison of *S. oralis* KE67213 *cps12* polysaccharide biosynthetic locus with the 17 gene pneumococcal *cps12F*. KE672-13 has a single orf (bases 12726–14978) that corresponds to the distinct pneumococcal orfs *wcxD* and *wcxE* as indicated. Otherwise, the two polysaccharides share exactly the same gene order. **(B)** Comparison of *S. oralis* KE66813 polysaccharide biosynthetic gene cluster with the 16 gene pneumococcal *cps15A*. The *wciZ* contains frameshift and is lacking altogether in strain KE66813. Although KE66813 contains a gene with homology to *wchX* that encodes a putative glycerol phosphotransferase, strain KE66813 lacks the three glycerol-2-phosphate synthesis genes (*gtp1*, *gtp2*, *gtp3*). **(C)** Comparison of *S. mitis* KE667-13 (“*cps18”*) polysaccharide biosynthetic locus with pneumococcal *cps18C* (accession CR931673). The asterisk for the pneumococcal *glf* gene indicates that it is a pseudogene that contains several stop codons, even though it is of the same exact length as its highly homologous *S. mitis* counterpart that contains no stop codons. The open reading frame *orf1* is highly homologous to an open reading frame in *S. mitis* SK667 (accession JPV01000015) while *orf2* has limited homology to *wcyF* (unknown function) within the pneumococcal *cps25A*, *cps25F*, and *cps38* operons. **(D)** Comparison of *S. oralis* KE66913 (“*cps33*”) polysaccharide biosynthetic gene cluster (orfs *wzg* through *glf*) with pneumococcal *cps33F* (GenBank accession CR931702). The open reading frame *wycO* (putative acetyltransferase) has indicated homology to the *cps21* homolog. Two full-length *aliB* (oligopeptide-binding protein) genes are indicated.
