# Supplementary figures and images for: Streptococcus infantis, Streptococcus mitis, and Streptococcus oralis Strains With Highly Similar cps5 Loci and Antigenic Relatedness to Serotype 5 Pneumococci
Source: Front Microbiol. 2019 Jan 8;9:3199. doi: 10.3389/fmicb.2018.03199 (PMC6332807; doi:10.3389/fmicb.2018.03199)

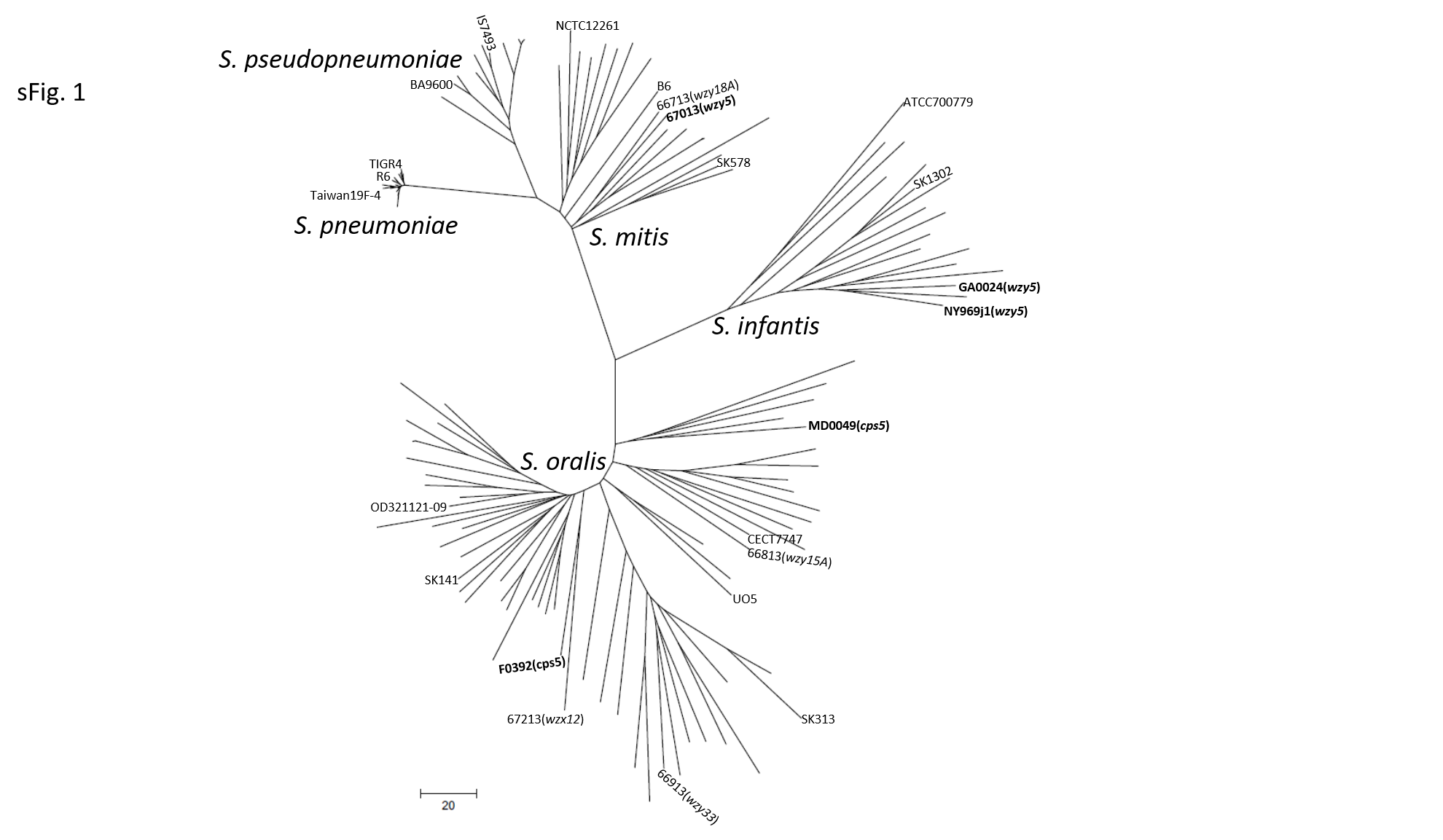

Supplement: FIGURE S1 — Assignment of study strains to species employing phylogenetic clustering of concatenated housekeeping genes as previously described (Bishop et al., 2009). Where not labeled the sequences were obtained from this study and shared the same species clustering. Methodology follows this paper exactly, except that only the four closest species clusters to S. pneumoniae were included. Twenty pneumococcal strains of 10 different serotypes were included. Established reference strains with genomes accessible in public databases include the indicated pneumococcal strains, ATCC BAA960 and IS7493 (S. pseudopneumoniae); NCTC 12261, B6, and SK578 (S. mitis); ATCC 700779, SK1302 (S. infantis); and CCECT7747, UO5, SK313, SK141, OD321121-09 (S. oralis). The optimal tree with the sum of branch length = 5152.37943912 is shown. The tree is drawn to scale, with branch lengths in the same units as those of the evolutionary distances used to infer the phylogenetic tree. The evolutionary distances are in the units of the number of base differences per sequence. The analysis involved 136 nucleotide sequences. Codon positions included were 1st+2nd+3rd+Non-coding. There were a total of 3063 positions in the final dataset for each strain. [file Image_1.TIF]

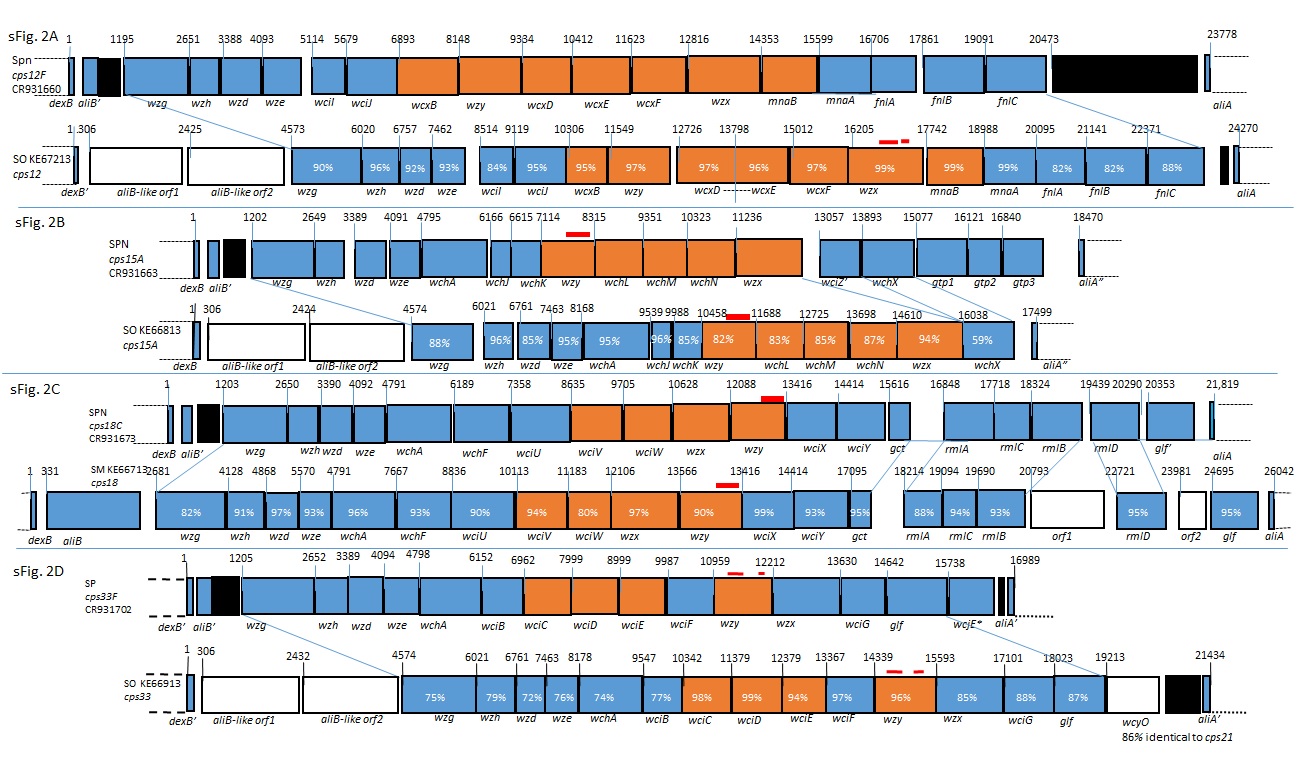

Supplement: FIGURE S2 — General features of additional homologs of cps loci from non-pneumococcal species (SO, S. oralis; SM, S. mitis) compared to pneumococci of known capsular serotypes. The genes aligned by the slanted lines indicate the polysaccharide synthetic gene cluster. Within each gene of the cluster, the percent identity between the two homologs is shown. In each cps operon shown the gene cluster lies between dexB and aliA. White rectangles indicate open reading frames (orfs) that lack homology with the respective pneumococcal reference sequence. The red lines indicate region targeted by conventional and real time “PCR-serotyping” assays. The orange open reading frames share >60% sequence identity only with pneumococcal strains within small serogroups as follows: A, 12F/12A/12B/44/46; B, 15A/15F; C, 18C/18A/18B/18F; D, 33F/33A/37. Black rectangles do not represent open reading frames, but have spurious homology to transposase structural genes. For descriptions of conserved and serotype-specific gene functions, see the indicated GenBank accessions for the pneumococcal reference serotypes as described (Bentley et al., 2006; Aanensen et al., 2007; Mavroidi et al., 2007). (A) Comparison of S. oralis KE67213 cps12 polysaccharide biosynthetic locus with the 17 gene pneumococcal cps12F. KE672-13 has a single orf (bases 12726–14978) that corresponds to the distinct pneumococcal orfs wcxD and wcxE as indicated. Otherwise, the two polysaccharides share exactly the same gene order. (B) Comparison of S. oralis KE66813 polysaccharide biosynthetic gene cluster with the 16 gene pneumococcal cps15A. The wciZ contains frameshift and is lacking altogether in strain KE66813. Although KE66813 contains a gene with homology to wchX that encodes a putative glycerol phosphotransferase, strain KE66813 lacks the three glycerol-2-phosphate synthesis genes (gtp1, gtp2, gtp3). (C) Comparison of S. mitis KE667-13 (“cps18”) polysaccharide biosynthetic locus with pneumococcal cps18C (accession CR931673). The a [file Image_2.JPEG]
